# Effectiveness of computed tomography perfusion imaging in stroke management

**DOI:** 10.3389/fneur.2024.1390501

**Published:** 2024-08-12

**Authors:** Martina Cviková, Michal Haršány, Jan Vinklárek, Jakub Štefela, Iva Fojtová, Robert Mikulík

**Affiliations:** ^1^Department of Neurology, Faculty of Medicine, St. Anne's University Hospital Brno, Masaryk University, Brno, Czechia; ^2^International Clinical Research Centre, St. Anne's University Hospital Brno, Brno, Czechia

**Keywords:** CT perfusion, stroke, stroke mimics, stroke imaging, acute management of stroke

## Abstract

**Objectives:**

Current guidelines do not support the use of computed tomography perfusion (CTP) in stroke, except when identifying the penumbra during an extended treatment window. Therefore, this study aimed to define the yield of CTP in diagnosing a stroke diagnosis beyond the imaging of the penumbra in the hyperacute phase (0–6 h) and an extended time window (6–24 h).

**Materials and methods:**

All consecutive patients with acute onset of symptoms within a 24-h window underwent CTP imaging. The diagnostic value of CTP was calculated against the clinical and radiological diagnoses of stroke. A positive CTP result was determined by the presence of either a core or penumbra on the RAPID summary. Clinical diagnoses corresponded to discharge diagnoses of stroke. A radiological diagnosis was established if early ischemic changes [Alberta Stroke Program Early CT Score (ASPECTS) <10] were observed on the baseline CT scan, acute infarction was confirmed on follow-up imaging, or symptomatic occlusion was evident on baseline CTA.

**Results:**

Between November 2018 and November 2019, 585 consecutive patients with an acute neurological deficit underwent multimodal CT imaging. A total of 500 patients (85%) were included, where 274 (55%) were within the hyperacute phase, 153 (31%) had a radiological diagnosis of stroke, and 122 (24%) had a clinical diagnosis of stroke. CTP yielded positive results only in patients with a confirmed stroke (positive predictive value and specificity of 100%). When CTP results were negative, 43% of the cases turned out to stroke mimics. Patients with stroke mimics were younger (66 ± 17 vs. 73 ± 13) and had lower scores on the National Institutes of Health Stroke Scale [median 0; interquartile range (IQR) 0–2 vs. median 4; IQR 2–6] compared to patients with CTP-negative strokes.

**Conclusion:**

In our study, CTP consistently indicated brain ischemia; therefore, in stroke management, CTP is most beneficial when it yields a positive result. A positive CTP result should prompt adequate stroke management actions without any delay. Conversely, a negative CTP result necessitates the consideration of both stroke and non-stroke diagnoses.

## Introduction

Computed tomography perfusion (CTP) has been established as a critical imaging technique for assessing the penumbra during interventions such as intravenous thrombolysis (IVT) and mechanical thrombectomy in an extended time window, i.e., ≥4h, 5h, or 6 h from symptom onset (1–4). CTP provides information not only about the presence of penumbra but also about specific types of brain ischemia. Such information could be helpful for stroke management, assisting not only in decision-making about acute treatments, such as intravenous thrombolysis (IVT), but also in overall patient care. At the time of admission, it is essential to know if a patient has experienced a stroke because such information is critical for patient management, which include decision-making about admission to the stroke unit, conducting dysphagia screening, managing blood pressure and temperature, among others. CTP might serve as an additional tool that can help in deciding whether a patient has a stroke or a stroke mimic.

The Czech Republic has one of the highest thrombolytic rates (with over 20% of ischemic stroke patients receive IVT) and one of the shortest door-to-needle times (DNT) for intravenous thrombolysis (with the median national door-to-needle times being approximately 20 min) (5). Although there are highly developed and effective stroke services and networks, many hospitals do not use CT perfusion imaging. The argument is that indications for mechanical thrombectomy are expanding, and patients in the later time window and with pervasive early ischemic changes are recommended for recanalization treatment (6, 7). However, the opinion from the centers that are using CTP is that CTP is changing clinical management not only because it provides penumbra imaging but also because it is increasing physicians' confidence in stroke diagnoses in general.

Therefore, this study aimed to explore the yield of CTP beyond the established and guidelines-supported recommendations. The primary goal of this study was to assess the accuracy of baseline CTP compared to a clinical stroke diagnosis in 0–6 h and 6–24 h time windows. To remove any subjectivity associated with a stroke diagnosis, we also compared CTP findings with radiological stroke diagnoses.

## Methods

We conducted a cohort study that included patients with a suspected acute ischemic stroke within both the hyperacute phase (0–6 h) and the extended time window (6–24 h) admitted to the Neurology Department, with an acute neurological deficit at the time of admission between November 2018 and November 2019.

### Patients

All patients who presented to the Neurology Department with an acute neurological deficit within 24 h of symptom onset underwent plain CT, CT angiography (CTA), and CTP as part of the routine protocol. Patients were referred to the CT facility either directly after the arrival of the ambulance at the hospital or from the outpatient unit of the Neurology Department, and less frequently, from the Emergency Department (5, 8). CTP was evaluated using RAPID software. Stroke onset was defined as the last time the patient was seen well, which also applied to wake-up strokes. Cases were identified through the RAPID software database and matched with patients in the hospital's electronic health record system using the variables such as date and time of CT perfusion, age, and sex. Another search utillized the ICD stroke code in the hospital's electronic health record system to identify all patients discharged with stroke diagnoses.

### Imaging protocol

The imaging protocol always includes CT, CTA, and CTP for all patients suspected of having a stroke within 24 h of symptom onset. If thrombolysis and/or thrombectomy are performed, follow-up CT is always performed 24–36 h after the treatment. If no thrombolysis and/or thrombectomy are performed, follow-up CT is usually performed but may be omitted in cases with a lack of clinical doubts or if a stroke mimic is suspected and diagnosed by other means, such as magnetic resonance imaging (MRI), electroencephalography (EEG), etc.

A multidetector scanner (120 kV, 328 mAs [419 mAs/slice], Brilliance iCT 256; Philips Healthcare, Cleveland, OH) was used. For non-contrast brain CT, the section thickness was set at 4.5 mm. For CT angiography studies, 60 ml of contrast agent (Iomeron 300; Mallinckrodt Pharmaceuticals) was injected via an 18-gauge intravenous cannula. CTP covered 80 mm of the brain from the basal ganglia level. Scanning began with a 5-s delay after injecting 40 ml of the contrast agent (1 scan every 1.8 s for 75 s). Imaging postprocessing was performed using automatic software (RAPID, iSchemaView). Images of poor quality, primarily due to severe motion artifacts, difficulties with volume measuring, or errors with contrast injection timing, were excluded from the assessment.

### Radiological and clinical diagnoses of stroke

The accuracy of baseline CTP in diagnosing brain infarction was primarily evaluated against the clinical diagnosis of stroke. A clinical diagnosis of stroke was established if a patient was discharged with a final diagnosis of stroke. The final diagnosis of stroke was determined based on the presence of neurological deficits, patient history, baseline and follow-up imaging findings, and other considerations, such as EEG if epileptic seizures are suspected, CSF analysis if needed, and other para-clinical methods. Then, the accuracy of baseline CTP was also compared to the radiological diagnosis of stroke to remove any potential subjectivity associated with a discharge stroke diagnosis. The radiological diagnosis of stroke was established if there were (a) early ischemic changes [Alberta Stroke Program Early CT Score (ASPECTS) <10] on baseline CT scan, (b) symptomatic large or peripheral vessel occlusion at baseline CTA, and/or (c) infarction on follow-up imaging. A radiologist evaluated all CT and MRI scans. CTP was considered positive if the core and/or penumbra was present on the RAPID summary; however, the positive CTP findings were considered artifacts, particularly those artificial findings located around the orbits and petrous bones, which do not align with vascular territories were not considered valid. Only default thresholds were used, indicating that the brain tissue with a cerebral blood flow under 30% was marked as the core, and the brain tissue with a Tmax over 6 s was considered critically hypoperfused. The penumbra was calculated as a mismatch volume between these two tissue volumes. The presence of infarction on follow-up imaging was regarded as new hypodensity on CT and/or diffusion restriction on MRI.

### Demographic and baseline variables

In this study, the following demographic and baseline variables were collected: age, sex, scores from the National Institute of Health Stroke Scale (NIHSS) score, patient history, the time of symptom onset, and the time of CT scan, CT findings (including the Alberta Stroke Program Early CT Score = ASPECTS, presence of vessel occlusion, core and penumbra presence on the RAPID summary), interventions, such as intravenous thrombolysis, endovascular treatment, modified thrombolysis in cerebral infarction (mTICI), presence of infarction on follow-up imaging, stroke etiology (TOAST classification), and modified Rankin scale. All data were anonymized.

## Statistics

Microsoft Excel version 2010 and SPSS statistics software were utilized for performing analyses. The results were expressed as means, medians, and percentages. The sensitivity, specificity, and positive and negative predictive values were calculated separately for clinical and radiological stroke diagnoses.

## Results

**Figure 1:**
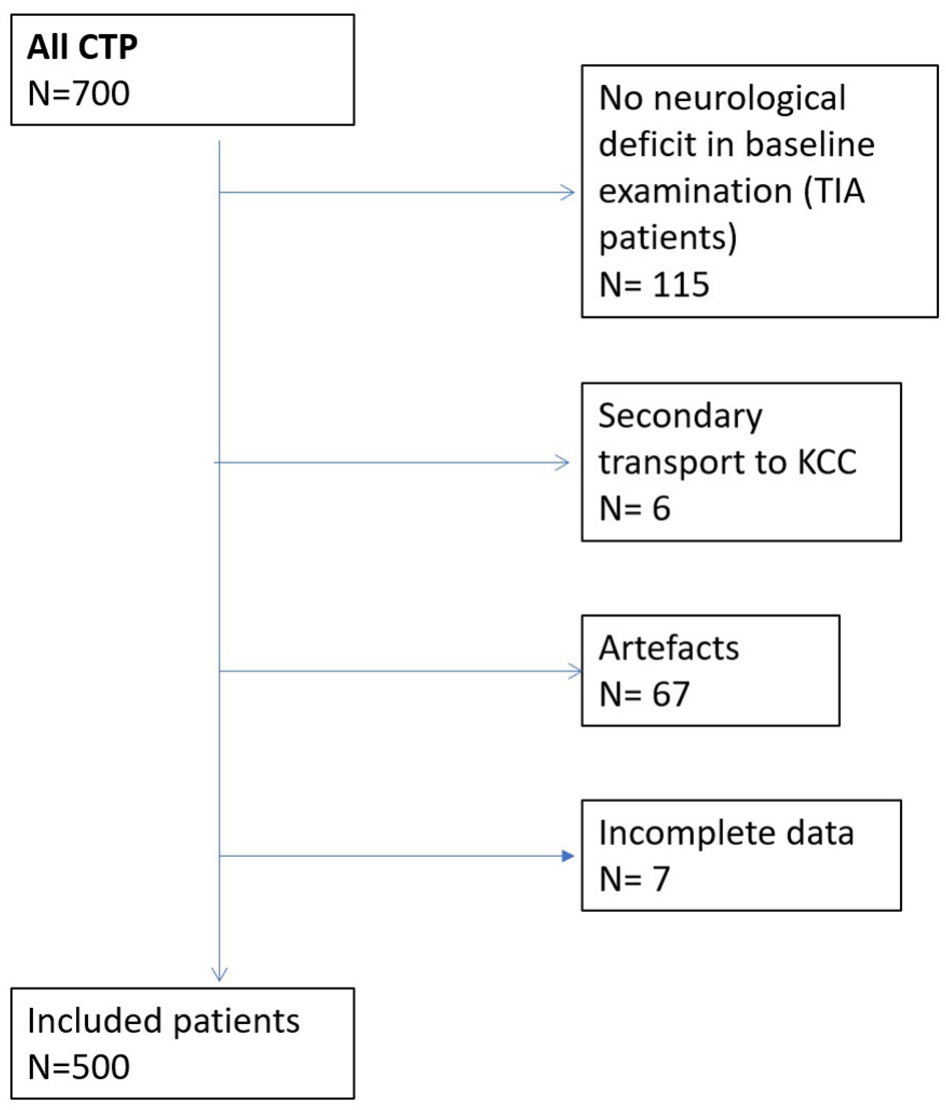
Flow diagram representing excluded patients.

From November 2018 to November 2019, 585 patients with acute neurological deficits underwent multimodal CT imaging. A total of 72 cases (12%) were excluded due to the CTP images were of poor quality or had severe motion artifacts. The study also excluded another six patients due to secondary transport to a comprehensive stroke center, and another seven patients were excluded due to incomplete data. The remaining 500 (85%) patients were included in the analysis: 232 (46%) patients in the 4 to 5-h thrombolytic window and 274 (55%) patients who had CTP in the 6-h window from the onset of symptoms. The average age was 71 ± 16 years, and 267 (53%) patients were female individuals. A flow diagram representing the excluded patients is shown in Figure 1. A total of 125 patients (25%) received intravenous thrombolysis, with a median door-to-needle time of 20 min. Table 1 shows all the baseline characteristics of the included patients.

**Table 1:** Baseline characteristics of both CTP-positive and CTP-negative patients.

	**All patients**	**CTP positive**	**CTP negative**
	***N** =* **500**	***N** =* **185**	***N** =* **315**
Age, mean ± SD	71 ± 16	73 ± 16	70 ± 15
**Sex**			
Female, *n* (%)	267 (53)	92 (50)	175 (56)
Baseline NIHSS median, (IQR)	4 (1–9)	10 (5–16)	2 (0–5)
NIHSS at discharge, median (IQR)	2 (0–5)	4 (2–9)	1 (0–3)
**Patient history**			
Diabetes mellitus, *n* (%; 95% CI)	154 (32; 28–36)	51 (28; 22–35)	108 (34; 29–40)
Arterial hypertension, *n* (%; 95% CI)	395 (80; 76–84)	152 (83; 77–88)	247 (78; 74–83)
Hyperlipidemia, *n* (%; 95% CI)	304 (61; 57–65)	121 (65; 58–72)	184 (58; 52–64)
Tobacco smoking, *n* (%; 95% CI)	96 (19; 16–23)	36 (20; 14–26)	63 (19; 15–24)
Atrial fibrillation/flutter, *n* (%; 95% CI)	150 (31; 27–35)	80 (44; 37–52)	73 (23; 19–28)
Stroke history, *n* (%, 95% CI)	138 (28; 24–32)	45 (25; 19–32)	94 (30; 25–35)
**Toast classification** ^*^			
1- atherothrombotic, *n* (%)	181 (36)	68 (37)	113 (36)
2- cardioembolic, *n* (%)	130 (26)	91 (49)	39 (12)
3- microangiopathy, *n* (%)	14 (3)	4 (2)	10 (3)
4- other known, *n* (%)	18 (4)	9 (5)	9 (3)
5- cryptogenic, *n* (%)	21 (4)	13 (7)	8 (3)
**Received treatment**			
Intravenous thrombolysis *n* (%)	125 (25)	97 (52)	28 (8)
Endovascular thrombectomy *n* (%)	56 (11)	56 (30)	0
**Imaging findings**			
ASPECTS median, (IQR)	10 (10–10)	10 (8–10)	10 (10–10)
Occlusion, *n* (%) ^*^	167 (33)	155 (85)	12 (3)
Extracranial occlusion, *n* (%) ^**^	10 (6)	10 (6)	0
Tandem occlusion, *n* (%) ^**^	18 (11)	18 (12)	0
Large intracranial occlusion, *n* (%) ^**^	64 (38)	62 (40)	2 (25)
Peripheral intracranial occlusion, *n* (%) ^**^	75 (45)	66 (42)	9 (75)
Infarction on follow up imaging, *n* (%)	180 (36)	87 (47)	93 (29)
**Final diagnosis**			
Stroke, *n* (%)	364 (73)	185 (100)	179 (57)
Stroke mimics, *n* (%)	136 (27)	0	136 (43)

SD, Standard Deviation; IQR, Interquartile Range; CI, Confidence Interval; ASPECTS, Alberta Stroke Program Early CT Score. ^*^TOAST classification relates only to 364 (73%) patients with diagnoses of stroke. ^**^Occlusion localization applies only to 167 (33%) patients with occlusion on CTA.

**Figure 2:**
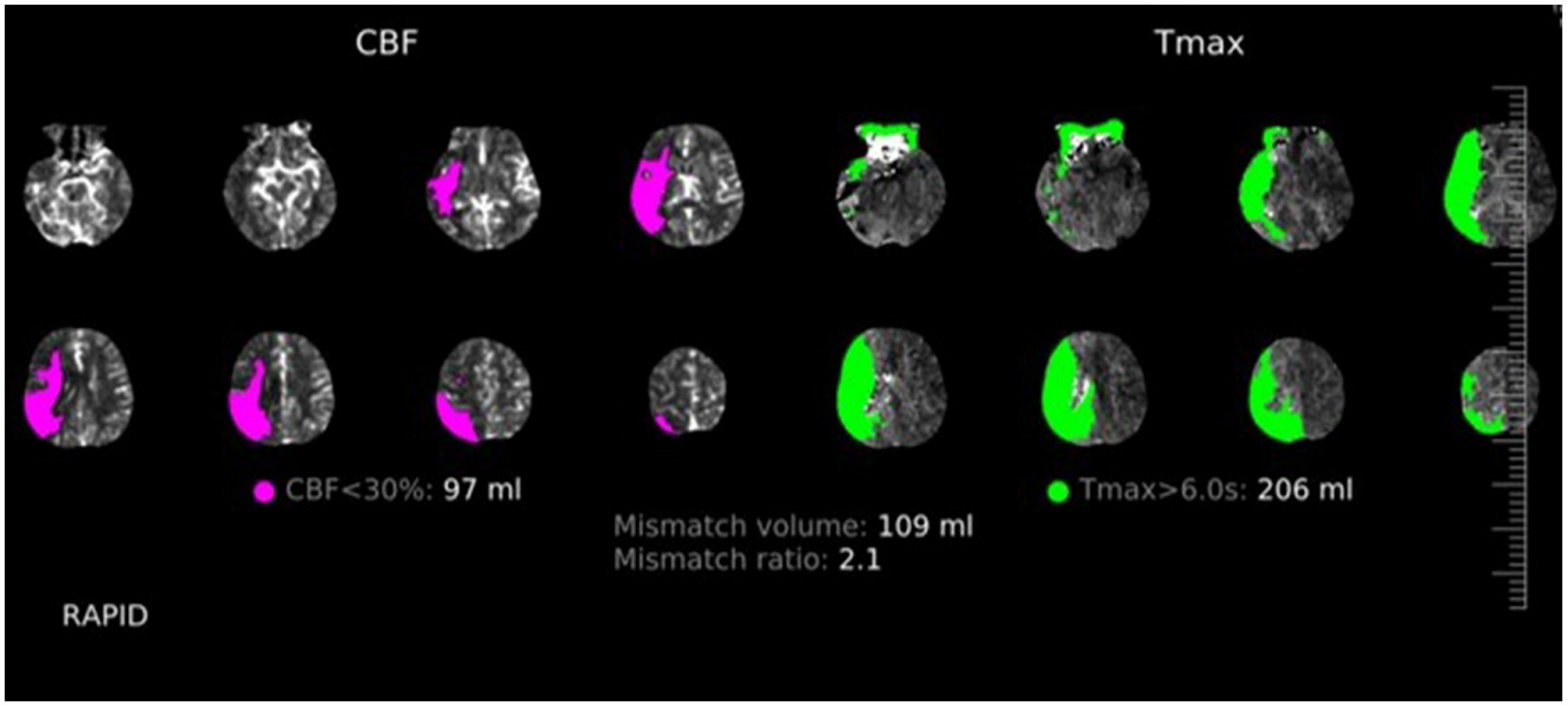
Flow diagram showing CTP findings, follow-up imaging, and definite diagnoses distribution in all patients.

**Figure 3:**
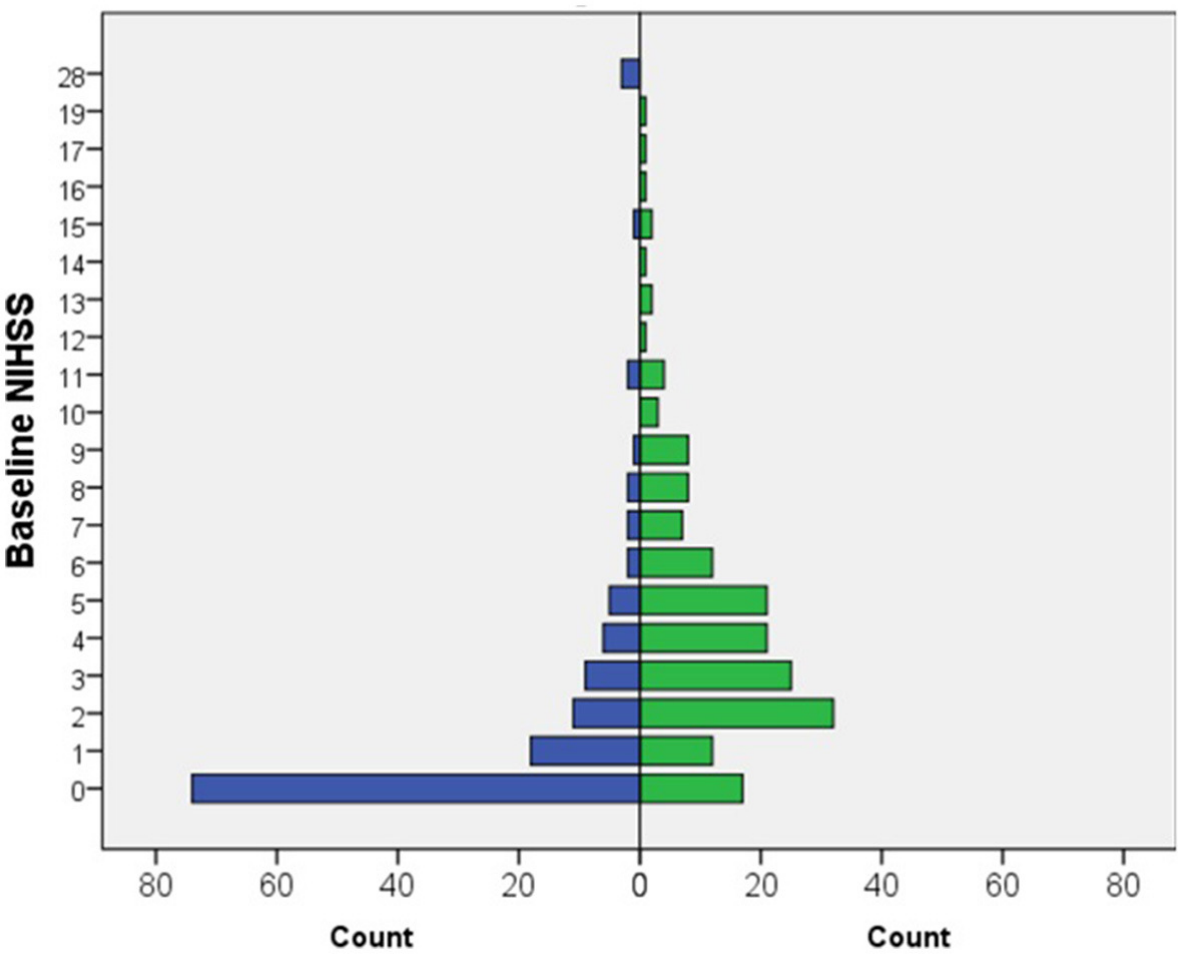
Bar chart documenting the distribution of NIHSS in patients admitted with a stroke suspicion.

Out of 500 patients, 185 (37%) had a positive CT perfusion was found in 185 patients (37%). A typical CTP pattern observed in a stroke patient is shown in Figure 2. All of these patients (100%) were dismissed with a final clinical diagnosis of stroke. Among the remaining 315 patients (63%) who showed negative initial brain perfusion, 179 (57%) had a clinical diagnosis of stroke, while the remaining 135 (43%) patients were identified as having conditions that mimin stroke. The overall sensitivity, specificity, and positive and negative predictive values of baseline CT for the clinical diagnosis of stroke were 51% [confidence interval (CI) 46–56], 100% (CI 97–100), 100% (CI 98–100), and 43% (CI 41–46), respectively. In the hyperacute phase (0–6 h from symptom onset), the sensitivity, specificity, and positive and negative predictive values of baseline CT for the clinical diagnosis of stroke were 53% (CI 46–60), 100% (CI 95–100), 100 (CI 97–100), and 41% (CI 38–45), respectively. The diagnoses of stroke mimics and their frequencies are shown in Table 2. In addition, the patient characteristics with the CTP-negative results, distinguishing between those with stroke mimics and strokes, are presented in Table 3, and a comparison of the patients in the hyperacute phase and extended time window is shown in Table 4. CTP-negative patients with stroke mimics were younger (66 ± 17 years vs. 73 ± 13 years) and had lower NIHSS scores [median 0, interquartile range (IQR) 0–2 vs. median 4, IQR 2–6] compared to CTP-negative patients with strokes. A bar chart demonstrating the distribution of NIHSS scores in patients admitted under the suspicion of stroke is shown in Figure 3.

**Table 2:** The prevalence of stroke mimics in our cohort was similar to a previously published systematic review (9).

	**Our cohort**	**Previously published systematic review (9)**
Stroke mimic; *n*	136	813
Vertigo of non-vascular cause; %	23	3
Epilepsy; %	18	20
Orthostatic collapse; %	12	12
Migraine; %	11	8
Amentia and delirium; %	7	2
Metabolic causes (hypoglycemia, hyponatremia, Wernicke encephalopathy); %	6	6
Hypertensive encephalopathy; %	5	1
Intoxications; %	4	2
Unknown cause; %	4	NA
Conversion disorders; %	2	7
Traumatic brain injury; %	1	0.5
Tumor; %	1	8
Plexopathy; %	1	5
Others (neuroinfection, traumatic SAH, post-hypoxic encephalopathy, impingement syndrome, ophthalmologic condition, and other extracerebral causes); %	7	NA

NA, not applicable.

**Table 3:** Baseline characteristics of stroke vs. stroke mimic patients.

	**All patients**	**CTP-negative stroke patients**	**CTP-negative stroke mimic patients**
	***N =* 315**	***N =* 179**	***N =* 136**
Age, mean ± SD	70 ±15	73 ± 13	66 ± 17
**Sex**			
Female, *n* (%)	175 (56)	92 (51)	83 (61)
Baseline, NIHSS, median (IQR)	2 (0–5)	4 (2–6)	0 (0–2)
**Patient history**			
Diabetes mellitus, *n* (%; 95% CI)	108 (34; 29–40)	63 (35; 28–43)	45 (33; 25–42)
Arterial hypertension, *n* (%; 95% CI)	247 (78; 74–83)	154 (86; 80–91)	93 (68; 60–76)
Hyperlipidemia, *n* (%; 95% CI)	184 (58; 52–64)	124 (69; 62–76)	60 (44; 36–53)
Tobacco smoking, *n* (%; 95% CI)	63 (19; 15–24)	45 (24; 18–31)	18 (13; 8–20)
Atrial fibrillation/flutter, *n* (%; 95% CI)	73 (23; 19–28)	44 (25; 19–32)	29 (21; 15–29)
Stroke history, *n* (%; 95% CI)	94 (30; 25–35)	57 (32; 25–39)	37 (27; 20–36)
NIHSS at discharge, median (IQR)	1 (0–3)	2 (0–3)	Not applicable
**Imaging findings**			
Occlusion, *n* (%)	12 (4)	12 (7)	0
Extracranial occlusion, *n*	1	1	0
Tandem occlusion, *n*	0	0	0
Large intracranial occlusion, *n*	2	2	0
Peripheral intracranial occlusion, *n*	9	9	0
Infarction on follow up imaging, *n* (%)	51 (16)	51 (28)	0
**Stroke localization**			
Lacunar supratentorial stroke (MRI confirmed), *n* (%)	24 (8)	24 (13)	Not applicable
Vertebrobasilar territory	66 (21)	66 (37)	Not applicable
Cortical stroke outside CTP coverage *n* (%)	10 (3)	9 (5)	Not applicable
Unknown *n* (%)	80 (25)	80 (45)	Not applicable
**Treatment**			
Intravenous thrombolysis *n* (%)	28 (8)	28 (15)	0 (0)
Door-to-needle time median (min)	20	20	Not applicable

**Figure 4:**
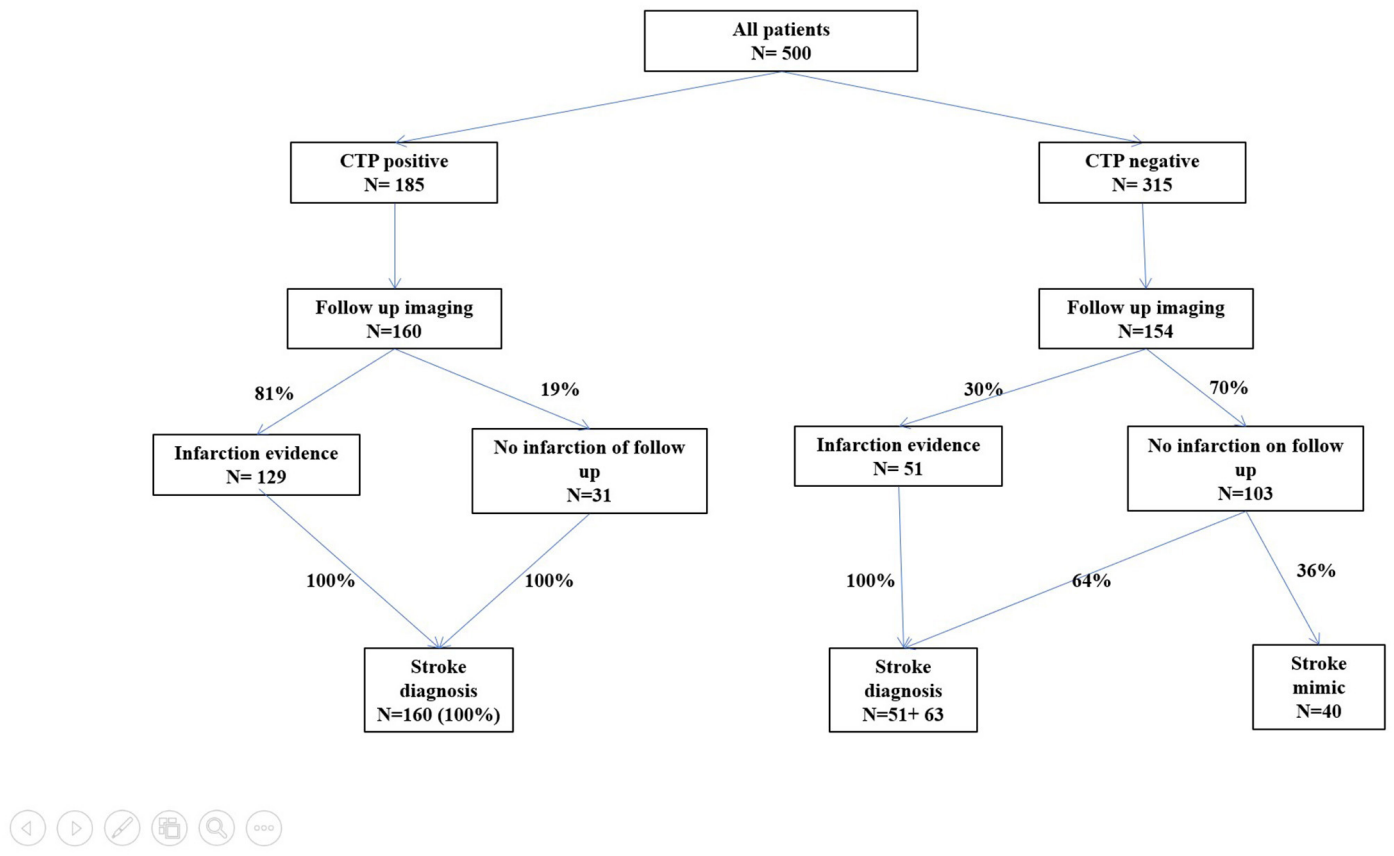
Typical CTP pattern in a stroke patient.

SD, Standard Deviation; IQR, Interquartile Range; CI, Confidence Interval. As all stroke mimic patients had negative-CTP results, they were compared to stroke patients who also had negative-CTP results. Patients with stroke mimics were younger and had lower NIHSS scores as compared to patients with stroke. See also Figure 4 for differences in the distribution of NIHSS between both groups.

**Table 4:** Documenting the main differences between patients with CTP performed in the hyperacute phase (0–6 h) and patients with CTP performed in the extended time window (6–24 h).

	**Patients in the hyperacute phase**	**Patients in the extended time window**
	***N** =* **274**	***N** =* **226**
**Patient characteristics**		
Age, mean ± SD	70 ± 15	72 ± 15
Female, *n* (%)	143	124
Baseline NIHSS median (IQR)	4 (0–8)	4 (0–8)
NIHSS at discharge median (IQR)	1 (0–2)	2 (0–5)
**Imaging findings**		
CTP positive *n* (%)	109 (40)	76 (34)
CTP negative *n* (%)	165 (60)	150 (66)
Detected vessel occlusion *n* (%)	102 (37)	65 (29)
**Treatment**		
Intravenous thrombolysis *n* (%)	96 (35)	29 (13)
Door-to-needle time median (min)	20	20
Mechanical thrombectomy *n* (%)	36 (13)	20 (9)
**Final diagnosis**		
Stroke *n* (%)	206 (75)	158 (70)
Stroke mimics *n* (%)	68 (25)	68 (30)

As expected, the thrombolytic and mechanical thrombectomy rates in the patients who underwent CTP during the hyperacute phase are significantly higher compared to those in the other group. The diagnosis distribution is similar in both groups.

A clinical (discharge) diagnosis of stroke in CTP-negative patients was established based on their clinical presentation and patient history in accordance with follow-up neuroimaging findings (*N* = 51), symptomatic vessel occlusion (*N* = 6), and significant symptomatic stenosis (*N* = 7). A total of 18 patients underwent intravenous thrombolysis, resulting in the resolution of symptoms and no infarction evident on the follow-up CT scan.

In patients who had negative initial CT perfusion with no follow-up imaging (*n* = 65), a definitive discharge diagnosis of stroke was determined based on the presence of symptomatic vessel stenosis or occlusion (*n* = 20), or evidence of infarction on the initial non-contrast CT scan (*n* = 5). In 46 out of 65 patients with negative initial CTP and no follow-up imaging, a stroke diagnosis was confirmed after the exclusion of stroke mimics. Most of these patients presented with lacunar syndrome.

Follow-up imaging (brain CT/MRI) was performed in 314 cases (63%). In 180 out of 314 cases (57%), new hypodensity during the CT scan was confirmed, and in 10 (5%) cases, the infarction was localized outside the area of the brain covered by baseline CTP. In CTP-positive patients, a radiological diagnosis of stroke was confirmed in 129 patients (81%), while in CTP-negative patients, 51 patients (33%) had infarction on follow-up imaging. CTP findings, follow-up imaging, and definite diagnosis distribution in all patients are shown in Figure 4. The overall sensitivity, specificity, and positive and negative predictive values of baseline CTP for the radiological diagnosis of stroke were 74% (CI 68–79), 94% (CI 88–97), 97% (CI 93–98), and 61% (CI 65–66), respectively. For patients with CTP within the 6 h of symptom onset, the sensitivity, specificity, positive and negative predictive values of baseline CTP for the radiological diagnosis of stroke were 80% (CI 72–86), 95 % (CI 86–99), 97% (CI 92–99), and 69% (CI 61–76), respectively.

## Discussion

In our study, all patients with an acute neurological deficit underwent CTP and 25% of them received IVT with a door-to-needle time of 20 min. Therefore, in the first place, our study documents the feasibility of conducting CTP as part of routine clinical practice. Such a finding has important clinical implications because some previous studies have demonstrated that CTP prolongs the initiation of IVT and thus should not be used (10). Our study documents that despite adding a few minutes for CTP imaging (usually 4–5 min), typically conducted in our center alongside unenhanced CT and CTA, CTP, in principle, does not pose a barrier to achieving very short DNT. The main reason is that patients are directly transported to undergo a CT scan from an ambulance, and IVT is initiated in a CT scanner, as we have published previously (7, 11).

The primary aim of our study was to compare the accuracy of baseline CTP to a clinical diagnosis of stroke. Our first major finding was that once CTP was positive, regardless of the time window, all patients had a stroke, and none were diagnosed with stroke mimics. Such information is beneficial because it eliminates any diagnostic doubts that physicians might have. Most likely, the value of stroke confirmation will be increased even more in those hospitals and countries where patients are not managed by stroke experts. Patients with positive CTP results should receive complete stroke care, which includes not only IVT but also admission to a stroke unit, management of blood pressure, and screening for dysphagia, among others, as per guidelines.

We were unable to confirm the results of some previous studies that have documented that hypoperfusion (and even hyperperfusion) on CTP imaging can also be found in patients with conditions other than stroke, such as status epilepticus or post-paroxysmal deficit (11–14). In our cohort, 23 patients had epileptic seizures, and all had negative CTP findings. The different results can be explained by the fact that in previous studies, no automatic software was used, no thresholds were defined, and the number of patients was low. As our study did not find any false positives, our results are applicable to clinical practice as long as certified postprocessing tools such as RAPID are used to evaluate CTP.

Our second major finding was that when CTP was negative, 57% had a final clinical diagnosis of stroke. When a radiological definition of stroke was used, 26% of CTP-negative patients suffered a stroke. False negatives were due to lacunar supratentorial infarction (24 patients) or vertebrobasilar stroke (66 patients). In addition, 10 infarcts were located in the brain area outside the coverage of baseline CTP. Our findings reflect the known limitations of brain perfusion imaging (15, 16). The calculated specificity of CTP against a radiological diagnosis of stroke (94%) is comparable to that reported in the most extensive published meta-analysis (17). We observed a numerically lower sensitivity (74%) compared to the radiological diagnosis, as reported in the previous largest and most recent analyses (80%) (17). If CTP covers the entire brain instead of a width of 8 cm, as in our study, the accuracy of CTP will slightly improve because it allows for the detection of infarctions in any part of the brain.

The low sensitivity and negative predictive value of CTP in a stroke can be effectively addressed by using MRI diffusion-weighted images (DWIs) in the acute phase of a stroke. In recent years, there has been a noticeable shift toward replacing CT with short 6–8-min MRI protocols. MRI demonstrates remarkably high sensitivity over 97% even in the initial minutes of a stroke (18–21). One of the most significant advantages of using MRI in clinical practice is its ability to reduce the incidence of thrombolysis stroke mimics by 50% (19). However, this approach is not without its drawbacks. The main disadvantages include the inferiority of MR angiography in vessel occlusion detection, typically distal, and the overestimation of the stenosis degree (20). These drawbacks should be carefully considered when making treatment decisions. Other disadvantages include a door-to-imaging time delay (a few minutes), the need for a second acute imaging modality for diagnostic or therapeutic reasons, higher costs, and MRI contraindications (e.g., recent vascular stents and pacemakers) (19, 21).

However, the remaining 43% of the patients with negative CTP were diagnosed with stroke mimics, which is quite a substantial number. These patients were discharged with another diagnosis, such as vertigo of non-vascular cause (23%), epilepsy (18%), orthostatic collapse, or others, as described in Table 2. Negative CTP-patients with stroke mimics were younger (68 years vs. 73 years, *p* < 0.001) and had lower NIHSS scores (0 vs. 4, *p* < 0.001) compared to negative CTP-patients with a stroke. Patients with stroke mimics also had fewer cardiovascular risk factors, most likely, due to younger age. The prevalence of stroke mimics in our study was 27%, closely aligning with the findings from the meta-analysis of 29 studies documenting a 26% prevalence of stroke mimics (9, 10). In addition, the diagnoses of stroke mimics were similar to those reported in the published data, as shown in Table 2. Therefore, our study documented a similar number and causes of stroke mimics. None of the patients with stroke mimics received IVT. We suggest that this is mainly due to the low NIHSS score and minor non-disabling deficits (median NIHSS 0 IQR 0–2). We found that if CTP is negative, it should raise a red flag in younger patients with minor neurological deficits and fewer cardiovascular risk factors because they might have stroke mimics. In cases where a patients presents with lacunar syndrome or there is suspicion of a stroke in the vertebrobasilar territory, negative CTP should not prevent treatment with intravenous thrombolysis.

One of the significant limitations of our study is the retrospective design. In a prospectively conducted study, CTP could be compared to the clinician's interpretation of the patient in the acute situation and non-contrast CT to assess the real added value of brain perfusion as a part of the standard imaging protocol. The other major limitation is the missing follow-up imaging in 37% of the cases. Such missing cases could limit conclusions about CTP accuracy against radiological diagnosis but not clinical stroke diagnosis. In our clinical practice, follow-up CT is omitted only when stroke or stroke mimic diagnoses are established. In clinically uncertain cases, we always perform an MRI. Therefore, missing follow-up imaging should not limit the validity of our study, although the confirmation of our results by conducting future studies will be reassuring. Another limitation of our study is that we excluded 12% of the patients with poor-quality CTP; such patients were excluded because they could not be assigned to either group (CTP positive or CTP negative). This exclusion of patients may have altered the specificity calculations, although the number of excluded patients is similar to other previously published studies (22–24). As mentioned earlier, our calculated specificity is comparable to the biggest published meta-analyses (17). Finally, although our results support the use of CTP in stroke management, we did not consider the economic cost in our study.

For the generalizability of our results, especially regarding the high proportion of stroke mimics, it might be essential to consider the referral pattern applied in our hospital, as described in the Methods section. Hospitals with different referral patterns may have fewer stroke mimics, mainly if some triage criteria are applied before imaging, and these hospitals might only refer the most apparent stroke candidates for CT scans. Our results related to stroke mimics (negative predictive value) are thus generalizable to unselected patients with acute neurological deficits. Our results related to the 100% positive predictive value of a stroke should be, however, applicable to any hospital regardless of the referral pattern.

The major strength of our study is the consistency in the use of multimodal imaging, which is not limited to any patient subgroup. This is the advantage over previous studies that included only patients with a stroke in anterior circulation (25), patients in a short time window (23), or patients with MRI follow-up imaging alone (24). Another advantage of our study is the relatively large number of patients, which was much less (usually up to 120 cases only) in previous studies (25–28).

## Conclusion

There are several new essential findings in our study. First, we documented the feasibility of using CTP in routine clinical practice without negatively impacting stroke logistics, measured as the door-to-needle time for intravenous thrombolysis. Second, we documented the benefit of CTP beyond the guidelines recommended application, which is currently penumbra imaging ≥4, 5, and 6 h from symptom onset. Our study revealed the usefulness of CTP as a diagnostic tool, even in the hyperacute phase of a stroke. Positive CTP results improve physicians' confidence in diagnosing a stroke and rules out stroke mimics. However, negative CTP results should raise concerns about non-stroke diagnoses, especially in younger patients with minor deficits and non-lacunar syndrome. Negative CTP results should not prevent the IVT treatment.

## Data availability statement

The raw data supporting the conclusions of this article will be made available by the authors, without undue reservation.

## Ethics statement

The studies involving humans were approved by the Ethics Committee of St. Anne's University Hospital in Brno. The studies were conducted in accordance with the local legislation and institutional requirements. The Ethics Committee/institutional review board waived the requirement of written informed consent for participation from the participants or the participants' legal guardians/next of kin due to the observational and retrospective nature of the study.

## Author contributions

MC: Conceptualization, Formal analysis, Writing – original draft, Writing – review & editing. MH: Data curation, Formal analysis, Methodology, Writing – review & editing. JV: Formal analysis, Methodology, Resources, Writing – review & editing. JŠ: Conceptualization, Investigation, Resources, Writing – review & editing. IF: Data curation, Formal analysis, Writing – review & editing. RM: Conceptualization, Funding acquisition, Supervision, Validation, Writing – review & editing.
